# Obesity – a risk factor for postoperative complications in general surgery?

**DOI:** 10.1186/s12871-015-0096-7

**Published:** 2015-07-31

**Authors:** Elke E.K.M. Tjeertes, Sanne S.E. Hoeks, Sabine S.B.J.C. Beks, Tabita T.M. Valentijn, Anton A.G.M. Hoofwijk, Robert Jan R.J. Stolker

**Affiliations:** 1 Department of anesthesiology, Erasmus University Medical Centre, Room H-1273, PO Box 2040, 3000, CA Rotterdam, The Netherlands; 2 Department of surgery, Orbis Medical Centre, Sittard, The Netherlands

**Keywords:** Obesity, Postoperative complications, Long-term survival, General surgery

## Abstract

**Background:**

Obesity is generally believed to be a risk factor for the development of postoperative complications. Although being obese is associated with medical hazards, recent literature shows no convincing data to support this assumption. Moreover a paradox between body mass index and survival is described. This study was designed to determine influence of body mass index on postoperative complications and long-term survival after surgery.

**Methods:**

A single-centre prospective analysis of postoperative complications in 4293 patients undergoing general surgery was conducted, with a median follow-up time of 6.3 years. We analyzed the impact of bodyweight on postoperative morbidity and mortality, using univariate and multivariate regression models.

**Results:**

The obese had more concomitant diseases, increased risk of wound infection, greater intraoperative blood loss and a longer operation time. Being underweight was associated with a higher risk of complications, although not significant in adjusted analysis. Multivariate regression analysis demonstrated that underweight patients had worse outcome (HR 2.1; 95 % CI 1.4-3.0), whereas being overweight (HR 0.6; 95 % CI 0.5–0.8) or obese (HR 0.7; 95 % CI 0.6–0.9) was associated with improved survival.

**Conclusion:**

Obesity alone is a significant risk factor for wound infection, more surgical blood loss and a longer operation time. Being obese is associated with improved long-term survival, validating the obesity paradox. We also found that complication and mortality rates are significantly worse for underweight patients. Our findings suggest that a tendency to regard obesity as a major risk factor in general surgery is not justified. It is the underweight patient who is most at risk of major postoperative complications, including long-term mortality.

**Electronic supplementary material:**

The online version of this article (doi:10.1186/s12871-015-0096-7) contains supplementary material, which is available to authorized users.

## Background

According to the World Health Organization, obesity has doubled since 1980, with a prevalence that is continuing to rise. In the United States, more than one-third of the adult population is currently obese [1]. As in Europe, obesity has also reached epidemic proportions, although with considerable geographic variation [2].

Being obese is associated with increased risk of a number of medical conditions, including diabetes, coronary artery disease, hypertension, hyperlipidemia and certain types of cancer [3]. Obesity reduces quality of life [4] and life expectancy itself [5–7]. However, recent studies show that, except for wound infections, complication rates are not increased in this group of patients [8–10]. Despite considerable investigation, the effect of different weight categories on all other types of postoperative complications and long-term survival remains controversial.

More recently a paradox between body mass index and survival is described in both cardiac and non-cardiac surgical population [11–13]. This paradox shows an inverse relationship between body mass index and mortality, with lower mortality rates among the overweight and mild obese and increased mortality rates in the underweight population.

We hypothesized that a tendency to consider obesity as a major risk factor in general surgery, is not justified. Therefore, this study was designed to determine influence of body mass index on postoperative complications and long-term survival after surgery.

## Methods

### Study sample

This study is a single-centre prospective analysis of postoperative complications in patients undergoing general surgery. We obtained data from all consecutive patients undergoing general surgery at our institution from March 2005 to December 2006. Since the beginning of 2005 this general teaching hospital contains a highly modern degree of automation and a reliable registration of the electronic medical record. All patients undergoing elective or urgent surgery within the mentioned study period were included. Exclusion criteria were procedures performed under local anesthesia, patients younger than 14 years old and assisting surgery for a specialism other than the surgery department (for example: a member of the surgical staff assisting in a gynecologic procedure). Bariatric surgery was not performed in this medical center. The study cohort consisted of 5030 procedures in 4479 patients. Because one of our primary endpoints is long-term survival, we decided to restrict our analyses to the patient’s first operation only. When a patient needed repeated surgery during the same hospital stay, we did include the need for a reoperation as a separate outcome measure. Patients (*n* = 186) of whom height or weight were not available were excluded. Therefore, the study population consisted of 4293 patients. The study complies with the Helsinki statement on research ethics and due to the non-interventional character of this study; approval by the medical ethical committee at time of enrolment was not necessary according to Dutch law. Even though, the local medical ethical committee granted a formal statement of approval retrospectively.

### Baseline characteristics

Before surgery all patients were seen by a surgeon or a surgical resident who collected the patient characteristics. Information was gathered about the patient’s medical history such as pulmonary, cardiac or cerebrovascular disease, American Society of Anesthesiologists (ASA) classification, diabetes, hypertension, any malignancy, medication, intoxications and height and bodyweight. Pulmonary disease was defined as any illness of the lungs or respiratory system, such as asthma, lung cancer, chronic infections, previous pulmonary embolisms, or chronic obstructive pulmonary disease (COPD). Cardiac disease refers to coronary artery disease with or without previous intervention, heart failure, arrhythmias, valvular heart disease or cardiomyopathy.

The Body Mass Index (BMI; kg/m^2^) was used, according to the recommendation of the World Health Organization, as the measure to classify underweight, overweight and obesity in adults.

Patients with a body mass index (BMI) > 30 kg/m^2^ were defined as obese and were compared to patients with underweight (BMI < 18.5 kg/m^2^), normal weight (BMI 18.5–25 kg/m^2^), and patients with overweight (BMI 25–30 kg/m^2^) [1]. Furthermore, we collected surgery related characteristics. Surgical risk was divided into low, intermediate and high-risk procedures as proposed by Boersma et al. in their surgical risk classification system [14]. Secondly, we collected the type of anaesthesia, divided into loco regional (i.e. neuraxial or peripheral nerve blocks) or general anaesthesia. Finally we determined whether the patient was treated in an inpatient or outpatient surgical setting.

### Postoperative and long-term outcome

Primary endpoints were complications within 30 days from surgery and long-term mortality. Patients were followed during hospital stay and during their visits to the outpatient clinic up to one year. To analyze the outcome we obtained the following data: length of hospital stay (LOS), blood loss, operating time and the presence of postoperative complications, e.g. wound infections, pneumonia, thromboembolic events, cardiovascular and cerebrovascular events, ICU-admission, readmission, the need for repeated surgery, as well as in-hospital mortality. For an objective interpretation of complications, we used a modified classification system proposed earlier by Clavien and Dindo, in order to increase uniformity in reporting outcome measures [15, 16]. Concisely, the grade of complications is based upon five grades, according to severity of the problem. Grade I is a minor and self-limiting complication, not needing any specific treatment. A grade II complication needs specific drug therapy (such as antibiotics), or a minor treatment such as opening the wound at the patient’s bedside, whereas a grade III complication needs invasive procedures such as percutaneous drainage of an abscess or repeated surgery. Grade IV are these complications with residual disability, including organ failure or resection. Finally grade V means the patient died due to his complications. Any event that deviated from a normal postoperative course was registered as a complication. Long-term survival was based on information from the national public register. All complications were independently graded by a surgical resident as well as a member of the surgical staff.

### Statistical analysis

We presented categorical variables as numbers and percentages. Continuous variables were presented as mean ± standard deviation (SD) when normally distributed, or as median and interquartile range (IQR) when data was skewed. A chi-square test was used for all categorical variables. Continuous variables were compared by using analysis of variance or the Kruskal Wallis test. In order to study the association between different BMI categories and postoperative complications, univariable and multivariable logistic regression models were used. Kaplan-Meier survival curves were calculated to assess the relation between the BMI categories and 5-year survival and compared with a log-rank test. The relation between BMI categories and long-term mortality was evaluated using multivariable Cox proportional hazard regression analysis. All potential confounders (age, gender, surgical risk, type of anesthesia, ASA classification, diabetes, hypertension, pulmonary -, cardiac -, or cerebrovascular disease and the presence of a malignancy) were entered in the multivariable model to ensure giving an unbiased as possible estimate in the regression models. Patients in different BMI categories were compared to those of normal weight. Results are reported as odds ratios (OR) or hazard ratios (HR) with a 95 % confidence interval. For all tests, significance was set at a two-sided *P*-value < 0.05. The statistical analyses were performed using SPSS, version 20.0.0 statistical software (SPSS Inc., Chicago, Illinois).

## Results

### Patient population

A total of 4293 patients were suitable for analysis, of which 1815 (42.3 %) were of normal weight, 100 (2.3 %) were underweight, 1635 patients (38.1 %) were overweight and 743 patients (17.3 %) were obese. Table 1 shows the baseline and surgery related characteristics of the study population.

**Table 1:** Baseline Characteristics

	Normal weight	Underweight	Overweight	Obese	*p* value
	BMI 18.5–25(kg/m2)	BMI < 18.5(kg/m2)	BMI 25–30(kg/m2)	BMI > 30(kg/m2)	
	(*N* = 1815)	(*N* = 100)	(*N* = 1635)	(*N* = 743)	
Demographics					
Age, years (mean ± SD)	53.7 (±18.9)	51.6 (±21.6)	57.0 (±15.5)^a^	55.5 (±14.9)^a^	<0.001
BMI (mean ± SD)	22.6 (±1.7)	17.3 (±1.1)	27.2 (±1.4)	33.5 (±3.4)	<0.001
Male sex (%)	893 (49.2 %)	39 (39.0 %)^a^	970 (59.3 %)^a^	315 (42.5 %)^a^	<0.001
**ASA classification (%)**		^a^	^a^	^a^	<0.001
I	727 (40.1 %)	31 (31.3 %)	535 (32.8 %)	135 (18.2 %)	
II	553 (30.5 %)	20 (20.2 %)	636 (39.0 %)	362 (48.8 %)	
III	460 (25.4 %)	39 (39.4 %)	412 (25.3 %)	223 (30.1 %)	
IV	72 (4.0 %)	8 (8.1 %)	47 (2.9 %)	21 (2.8 %)	
V	1 (<1 %)	1 (<1 %)	0 (0.0 %)	1 (<1 %)	
**Medical history (%)**					
Diabetes mellitus	86 (4.7 %)	6 (6.1 %)	162 (9.9 %)^a^	134 (18.1 %)^a^	<0.001
Hypertension	257 (14.2 %)	14 (14.1 %)	360 (22.1 %)^a^	225 (30.3 %)^a^	<0.001
Cerebrovascular disease	123 (6.8 %)	8 (8.1 %)	118 (7.2 %)	54 (7.3 %)	0.919
Malignant disease	451 (24.9 %)	25 (25.3 %)	362 (22.2 %)^a^	172 (23.2 %)	0.308
Pathological cardiac history	302 (16.7 %)	18 (18.2 %)	316 (19.4 %)^a^	158 (21.3 %)^a^	0.033
Pathological pulmonary history	261 (14.4 %)	15 (15.2 %)	205 (12.6 %)	138 (18.6 %)^a^	0.002
Current smoking^b^	490 (35.4 %)	39 (48.8 %)^a^	374 (30.4 %)^a^	163 (26.9 %)^a^	<0.001
**Surgery risk (%)**		^a^		^a^	<0.001
Low	1078 (59.4 %)	33 (33.0 %)	969 (59.3 %)	365 (49.1 %)	
Intermediate	643 (34.4 %)	52 (52.0 %)	577 (35.3 %)	350 (47.1 %)	
High	94 (5.2 %)	15 (15.0 %)	89 (5.4 %)	28 (3.8 %)	
**Type of anesthesia (%)**					
General	1499 (82.8 %)	93 (93.9 %)^a^	1376 (84.3 %)	684 (92.2 %)^a^	<0.001
**Surgical setting (%)**					
Outpatient surgery	690 (38.0 %)	22 (22.0 %)^a^	607 (37.1 %)	216 (29.1 %)^a^	<0.001

^a^Significantly different (*p* < .05) compared to normal weight

^b^Data was available in 76.9 % of patients

When categorized by BMI, obese patients had more comorbidities, such as diabetes (*P* < .001), hypertension (*P* < .001), cardiovascular disease (*P* = .006) and pulmonary disease (*P* = .010) than patients of normal weight. High-risk surgery was more often performed in the group of underweight patients (*n* = 15, 15.0 %), while in the obese group; the surgical risk was predominantly low or intermediate (*n* = 725, 96.2 %). Table 2 shows the use of cardiovascular and pulmonary medication at time of surgery.

**Table 2:** Baseline Characteristics; Medication

	Normal weight	Underweight	Overweight	Obese	*p* value
	BMI 18.5–25(kg/m2)	BMI < 18.5(kg/m2)	BMI 25–30(kg/m2)	BMI > 30(kg/m2)	
	(*N* = 1815)	(*N* = 100)	(*N* = 1635)	(*N* = 743)	
Medication groups					
Antiplatelet therapy	214 (11.8 %)	12 (12.0 %)	247 (15.1 %)^a^	122 (16.4 %)^a^	0.005
Anticoagulant therapy	59 (3.3 %)	5 (5.0 %)	62 (3.8 %)	35 (4.7 %)	0.31
ß­blockers	165 (9.1 %)	13 (13.0 %)	225 (13.8 %)^a^	116 (15.6 %)^a^	<0.001
Calcium channel blockers	66 (3.6 %)	2 (2.0 %)	80 (4.9 %)^a^	58 (7.8 %)^a^	<0.001
Angiotensin-converting enzyme inhibitors	103 (5.7 %)	4 (4.0 %)	123 (7.5 %)^a^	78 (10.5 %)^a^	<0.001
Angiotensin-II receptor antagonists	58 (3.2 %)	1 (1.0 %)	118 (7.2 %)^a^	72 (9.7 %)^a^	<0.001
Statins	195 (10.7 %)	10 (10.0 %)	238 (14.6 %)^a^	141 (19.0 %)^a^	<0.001
Diuretics	199 (11.0 %)	13 (13.0 %)	252 (15.4 %)^a^	147 (19.8 %)^a^	<0.001
Nitrates	90 (5.0 %)	3 (3.0 %)	119 (7.3 %)^a^	42 (5.7 %)	0.018
Pulmonary medication	86 (4.7 %)	4 (4.0 %)	71 (4.3 %)	48 (6.5 %)	0.153

^a^Significantly different (*p* < .05) compared to normal weight

### Postoperative complications

**Fig. 1:**
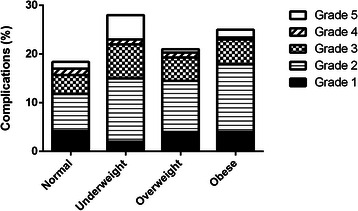
Bar Chart of Different Complication Grades

Obesity resulted in a longer operation time (*P* < 0.001), more intraoperative blood loss (*P* < 0.001) and higher rates of surgical site infections (*P* < 0.001) (Table 3). Underweight patients also had higher rates of complications than normal weight patients (Table 3). The overall mortality rate within 30 days was 1.2 % (52 patients), with a disadvantage for underweight patients (*n* = 4, 4.0 %). Complication grades were different between groups, with more non self-limiting (>grade 1) complications in the underweight (*n* = 25, 25 %), overweight (*n* = 277, 16.9 %) and the obese (*n* = 154, 20.7 %), compared to 14.2 % (*n* = 258) in normal weight patients (overall *P*-value *P* < 0.001) (Fig. 1 and Additional file 1).

**Table 3:** Postoperative Outcome within 30 Days

	Normal weight	Underweight	Overweight	Obese	*p* value
	BMI 18.5–25(kg/m2)	BMI < 18.5(kg/m2)	BMI 25–30(kg/m2)	BMI > 30(kg/m2)	
	(*N* = 1815)	(*N* = 100)	(*N* = 1635)	(*N* = 743)	
Wound infection	87 (4.8 %)	11 (11.0 %)^a^	127 (7.8 %)^a^	81 (10.9 %)^a^	*P* < 0.001
Pneumonia	31 (1.7 %)	4 (4.0 %)	41 (2.5 %)	16 (2.2 %)	*P* = 0.231
Deep vein thrombosis and/or pulmonary embolism	7 (0.4 %)	1 (1.0 %)	5 (0.3 %)	5 (0.7 %)	*P* = 0.474
ICU admission	232 (12.8 %)	27 (27.0 %)^a^	198 (12.1 %)	95 (12.8 %)	*P* < 0.001
Reoperation	87 (4.8 %)	11 (11.0 %)^a^	72 (4.4 %)	39 (5.2 %)	*P* = 0.028
Readmission	57 (3.1 %)	5 (5.0 %)	67 (4.1 %)	34 (4.6 %)	*P* = 0.246
Length of hospital stay (days) (median + IQR)	3 (1–8)	7 (3–16) ^a^	2 (1–7) ^a^	2 (1–7)	*P* < 0.001
Operation time (minutes) (median + IQR)	39 (24–65)	41 (27–90)	41 (26–66)	50 (27–80) ^a^	*P* < 0.001
Blood loss (mL)^b^ (median + IQR)	10 (5–50)	25 (5–138) ^a^	15 (5–50)	20 (10–100) ^a^	*P* < 0.001
30 days mortality	27 (1.5 %)	4 (4.0 %)	11 (0.7 %)^a^	10 (1.3 %)	*P* = 0.008
Cardiovascular complication	67 (3.7 %)	4 (4.0 %)	53 (3.2 %)	26 (3.5 %)	*P* = 0.897
Any complication	339 (18.7 %)	28 (28.0 %)	345 (21.1 %)	185 (24.9 %)	*P* = 0.001

^a^Significantly different (*p* < .05) compared to normal weight

^b^Data was available in 84.3 % of patients

A multivariate regression analysis, adjusting for confounders, demonstrated that obesity was associated with a higher risk of postoperative complications (OR 1.3; 95 % CI 1.1–1.7) (Table 4).

**Table 4:** Univariate and Multivariate Associations of BMI-categories and Complications/Mortality

		30 days complications		Long-term mortality		
BMI-categories	*N* (%)	OR (95 % CI)	Adjusted* OR (95 % CI)	*N* (%)	HR (95 % CI)	Adjusted* HR (95 % CI)
Normal weight BMI 18.5–25 (kg/m2)	334 (18.4)	1	1	331 (18.6)	1	1
Underweight BMI < 18.5 (kg/m2)	28 (28.0)	1.67 (1.05–2.63)	1.20 (0.73–1.97)	35 (35.4)	2.14 (1.51–3.05)	2.07 (1.44–2.96)
Overweight BMI 25–30 (kg/m2)	343 (21.0)	1.17 (0.99–1.38)	1.14 (0.95–1.36)	212 (13.2)	0.68 (0.58–0.81)	0.63 (0.53–0.75)
Obese BMI > 30(kg/m2)	186 (25.0)	1.46 (1.19–1.79)	1.31 (1.05–1.65)	109 (14.8)	0.77 (0.62–0.96)	0.71 (0.56–0.89)

^a^Potential confounders: age, gender, surgical risk, type of anesthesia, ASA classification, diabetes, hypertension, pulmonary -, cardiac - or cerebrovascular disease and the presence of malignancy

### Long-term survival

**Fig. 2:**
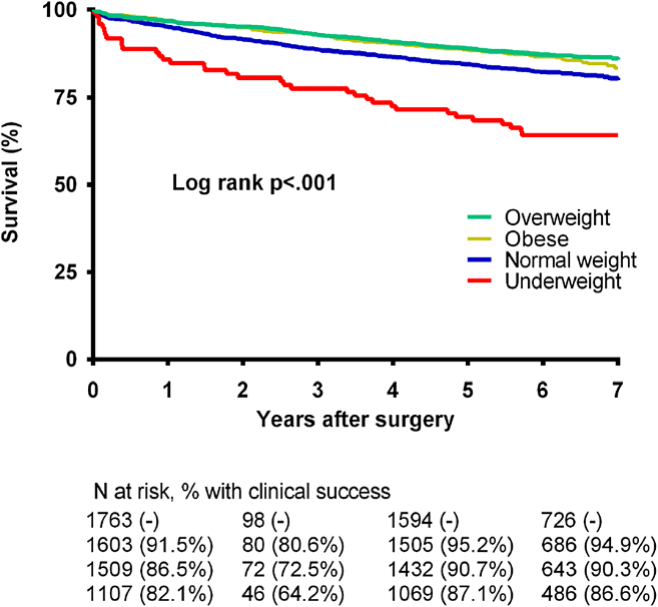
Kaplan Meier Estimate of Overall Long-term Survival

Long-term survival was based on information from the national public register, available in 4218 patients (98.3 %), with a median follow-up time of 6.3 (interquartile range 5.8–6.8) years. Last available follow-up information was used for 93 patients (2.2 %) who lived abroad or had emigrated. A total of 687 patients (16.3 %) died during a follow-up of 6.3 (IQR 5.8–6.8) years, including the 52 patients who died within 30 days of first hospital admission. Figure 2 shows a Kaplan-Meier estimate of overall long-term survival. Six year survival estimates varied significantly among the different BMI-categories: 64.2 % in the underweight group, 82.1 % in the normal weight group, 87.1 % in the overweight group and 86.6 % in the obese group. Multivariate regression analysis, adjusting for confounders, demonstrated that underweight patients undergoing general surgery again had the worst outcome (HR 2.1; 95 % CI 1.4–3.0), whereas being overweight (HR 0.6; 95 % CI 0.5–0.8) or obese (HR 0.7; 95 % CI 0.6–0.9) is associated with improved survival (Table 4).

## Discussion

In this large sample of patients we found that obesity is a significant risk factor for surgical site infection, more surgical blood loss and a longer operation time, however these complications did not affect long-term survival. Our finding that the incidence of surgical site infection increases with an increase of BMI confirms previous studies [8, 17–19]. A couple of explanations can be given for this association. First of all, excessive subcutaneous fat tissue predisposes these patients to impaired healing due to low regional perfusion and oxygen tension [20]. Secondly, in our study there was an increase in operation time for the obese and a longer operation time has been described as a significant predictor of postoperative wound infections [17, 18]. Furthermore impaired immunity, elevated blood glucose levels and too much tension on the surgical incision are also contributory factors to impaired wound healing [21, 22]. Thus, with exception of the complications described earlier, there was no difference in risk of any major postoperative adverse event between the obese and patients of normal weight. Being overweight or obese was actually associated with improved 30 days and long-term survival, also known as the obesity paradox. Increased awareness of both the surgeon and the anesthesiologist of obesity related health hazards might have contributed to improved perioperative care [23, 24]. Another explanation could be that obese patients are less often referred for major surgery, leading to election bias.

When compared to patients of normal weight, the underweight patients had a higher ASA classification and a higher risk of postoperative complications. It should be noted however that the underweight patients represent a rather small number of the total study population and results, especially short-term complications, should be interpreted with caution. In the present study, a bigger proportion of patients who underwent high-risk surgery were underweight, although not statistically significant. The underweight group contained more smokers, a potential confounder, since smoking is associated with wound infection, weight loss and chronic diseases [25, 26]. Also recent weight loss of more then 10 % or low serum albumin levels are known predictors of postoperative morbidity and mortality [27–29]. With the hypothesis that cachexia might be related to an unhealthy lifestyle or non-compliance, we compared the use of medication between the different BMI groups. We conclude that there was no undertreatment of pulmonary or cardiovascular medication in the underweight group. Unlike we expected, the incidence of malignant disease was not different between underweight and normal weight patients, which might again be explained by a relatively small sample size of the underweight group. Besides complications, we focused on postoperative mortality and long-term prognosis. Our study supports recent data and shows a significantly higher mortality rate for the lowest of BMI rankings [30].

This study has a few potential limitations that must be addressed. First, the recorded data on height and weight were partially self-reported, although this can be considered as a reliable estimate of BMI [31]. There might be a bias in referral pattern, since patients with major comorbidities and the super obese are usually seen in a tertiary hospital. With the prevalence of obesity in our study population being almost twice as high as in the Dutch population, this might not be an important bias [2]. Furthermore, we restricted analyses to patient’s first operation. Repeated surgery within the study period was often performed because of the same illness; for example a sentinel node procedure, followed by a mastectomy in the next hospital stay. A sensitivity analysis showed no difference in crude or adjusted estimates when including all duplicate cases. We did not have a direct measurement of central (or visceral) adiposity. Instead we used BMI as an indicator of adiposity, but the BMI is unable to distinguish between different kinds of body mass [32, 33].

The surgical procedures in this study have been performed eight up to nine years ago. Advances in clinical medicine can alter current practice. Finally, due to the observational character, this study is inherent to unmeasured confounding.

## Conclusion

In conclusion, our findings suggest that a tendency to consider obesity as a major risk factor in general surgery is not justified. It is the underweight patient who is most at risk of major postoperative complications, including long-term mortality.

## Additional file

Additional file 1: **Resume table with complications divided in different complication groups.** (DOCX 52 kb)

## Abbreviations used

ASA: American Society of Anesthesiologists
BMI: Body mass index
COPD: Chronic obstructive pulmonary disease
HR: Hazard ratio
ICU: Intensive care unit
IQR: Interquartile range
LOS: Length of hospital stay
OR: Odds ratio
SD: Standard deviation
