# Erratum to: Obesity – a risk factor for postoperative complications in general surgery?

**DOI:** 10.1186/s12871-015-0136-3

**Published:** 2015-10-26

**Authors:** E. K. M. Tjeertes, S. E. Hoeks, S. B. J. C. Beks, T. M. Valentijn, A. G. M. Hoofwijk, R. J. Stolker

**Affiliations:** 1 Department of anesthesiology, Erasmus University Medical Centre, Room H-1273, Rotterdam, CA The Netherlands; 2 Department of surgery, Orbis Medical Centre, Sittard, The Netherlands

Unfortunately the original version of this article [1] contained a mistake. The author list contained superfluous initials for each author. The corrected author list can be found below:

E.K.M. Tjeertes, S.E. Hoeks, S.B.J.C. Beks, T.M. Valentijn, A.G.M. Hoofwijk and R.J. Stolker
