# Supplementary material for: Obesity – a risk factor for postoperative complications in general surgery?
Source: BMC Anesthesiol. 2015 Jul 31;15:112. doi: 10.1186/s12871-015-0096-7 (PMC4520073; doi:10.1186/s12871-015-0096-7)
Supplement: Additional file 1: — Resume table with complications divided in different complication groups. (DOCX 52 kb) [file 12871_2015_96_MOESM1_ESM.docx]

**Resume table with complications divided in different complication groups**

|  | No complications % | Self-limiting complications  (Grade 1) % | Non self-limiting complications  (Grade 2+3) % | Major complications  (Grade 4+5) % |
| --- | --- | --- | --- | --- |
| Normal weight | 1480 (81.5%) | 77 (4.2) | 209 (11.5) | 49 (2.7) |
| Underweight | 73 (73.0%) | 2 (2.0) | 20 (20.0) | 5 (5.0) |
| Overweight | 1293 (79.1%) | 65 (4.0) | 249 (15.2) | 28 (1.7) |
| Obese | 559 (75.2%) | 30 (4.0) | 141 (19.0) | 13 (1.7) |
